# Supplementary material for: Association between oral microbiome and seven types of cancers in East Asian population: a two-sample Mendelian randomization analysis
Source: Front Mol Biosci. 2023 Nov 21;10:1327893. doi: 10.3389/fmolb.2023.1327893 (PMC10702768; doi:10.3389/fmolb.2023.1327893)
Supplement: Supplementary file 1 [file DataSheet1.ZIP › Supplementary Materials/MR plots for tongue/tongue═╝/Gastric cancer/pheno.1215_to_gastric cancer_forest.pdf]

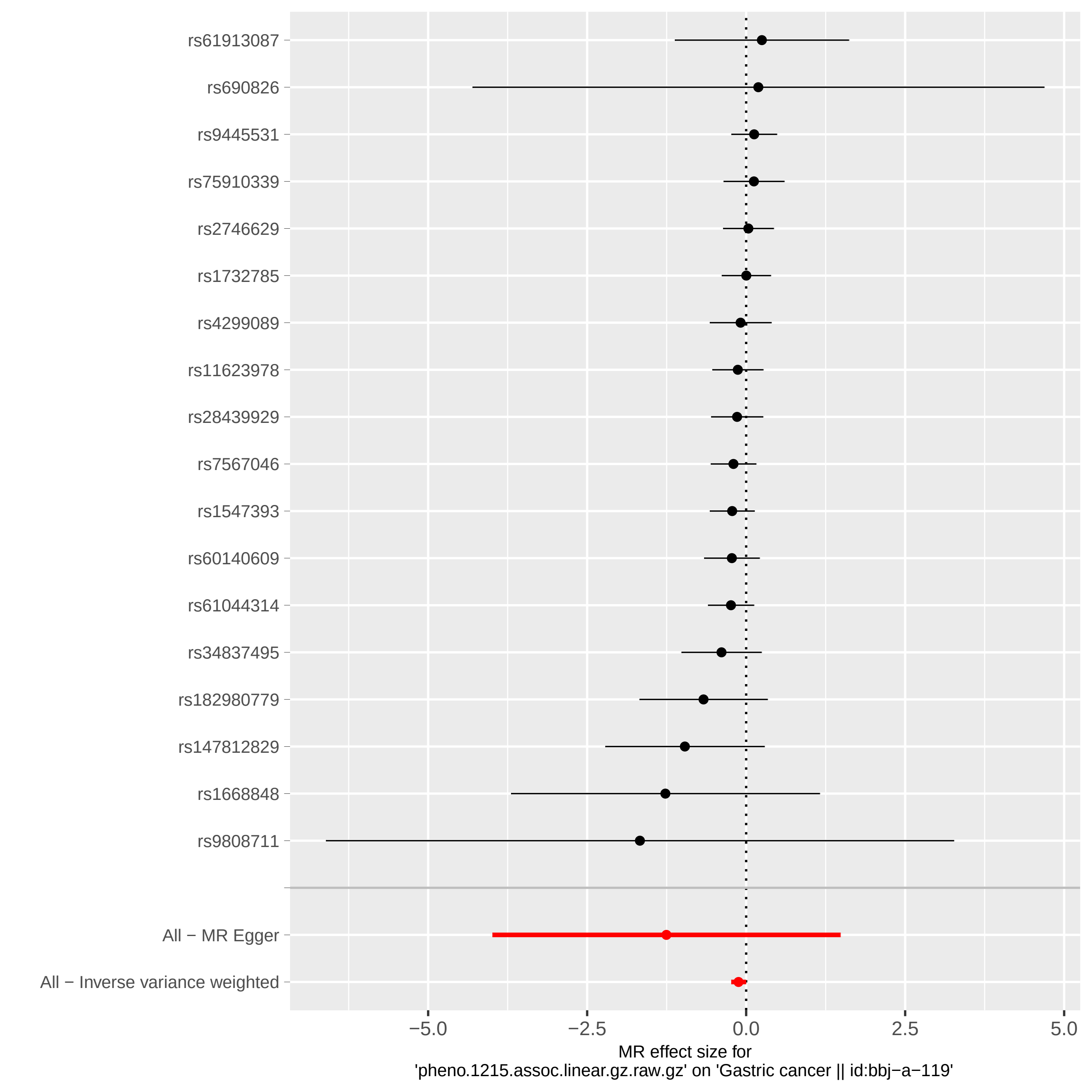
rs61913087
rs690826
rs9445531
rs75910339
rs2746629
rs1732785
rs4299089
rs11623978
rs28439929
rs7567046
rs1547393
rs60140609
rs61044314
rs34837495
rs182980779
rs147812829
rs1668848
rs9808711
All − MR Egger
All − Inverse variance weighted
−5.0
−2.5
0.0
2.5
5.0
MR effect size for
'pheno.1215.assoc.linear.gz.raw.gz' on 'Gastric cancer || id:bbj−a−119'
